# Low-Cost–High-Efficacy Control of Faba Bean Aphids by Fungal and Chemical Insecticides Co-Applied at Low and Sublethal Rates

**DOI:** 10.3390/insects16060565

**Published:** 2025-05-27

**Authors:** Sen-Miao Tong, Dan-Yi Qi, Xin-Yue Tang, Yan-Zhi Shao, Shi-Tong Hu, Yu-Piao Zheng, Xin-Yun Zheng, Ling-Li Yan, Ming-Guang Feng

**Affiliations:** 1 College of Advanced Agricultural Sciences, Zhejiang A&F University, Hangzhou 311300, China; 2 Institute of Microbiology, College of Life Sciences, Zhejiang University, Hangzhou 310058, China

**Keywords:** *Beauveria bassiana*, *Aphis fabae*, fungal–chemical interaction, aphid biocontrol

## Abstract

The high costs of mycoinsecticides restrain their extensive application in green agriculture. Two six-week field trials were performed in spring to test synergistic effects of fungal–chemical interactions against faba bean aphid (*Aphis fabae*) populations in Zhejiang, China. The treatments (three 50-m^2^ plots each) in each trial included *Beauveria bassiana* ZJU435 (Bb) and imidacloprid (ImD) applied biweekly at recommended rates (1.5 × 10^13^ conidia and 45 a.i. g ha^−1^) and co-applied at reduced rates of 2/3Bb, 1/2Bb, and 1/3Bb plus 1/5ImD, which reduced the cost of the mycoinsecticide by 32–65%. During the first three weeks at 1.3–12.5 °C (daily means), the aphid population steadily increased to a peak in the blank control, and it was much more effectively suppressed by ImD than by Bb and Bb + ImD treatments. As the weather subsequently warmed to 8.7–21.0 °C, the aphid population was increasingly suppressed by the Bb and Bb + ImD treatments, which showed suppressive effects superior or close to those of ImD on days 35 and 42. Percent efficacy values (*n* = 36) of ImD, 2/3Bb + 1/5ImD, 1/2Bb + 1/5ImD, Bb, and 1/3Bb + 1/5ImD in two six-week trials averaged respectively 86%, 66%, 62.2%, 59.4%, and 58.4%, which significantly differed from one another (*p* < 0.01). Conclusively, low-rate co-applications of fungal/chemical insecticides offer a ‘low-cost–high-efficacy’ strategy to promote extensive mycoinsecticide application for sustainable aphid control.

## Simple Summary

The production costs of environmentally friendly fungal insecticides are much higher than those of their conventional chemical counterparts, hindering their extensive application. Synergistic effects of fungal–chemical interactions against insect pests can be exploited to enhance fungal efficacy at reduced application cost but still lack direct field evidence for support. Our field data confirm that the mycoinsecticide *Beauveria bassiana* ZJU435 applied at 2/3, 1/2 and 1/3 of its recommended rate plus 1/5 of imidacloprid label rate was significantly more or equally efficacious against faba bean aphids than the fungus was when applied at its recommended rate. The fungal–chemical combinations reduced the fungal application cost by 32–65%, supporting a ‘low-cost–high-efficacy’ strategy to promote extensive mycoinsecticide application for sustainable aphid control and to mitigate chemical side effects on agroecosystems and environments.

## 1. Introduction

The black bean aphid *Aphis fabae* L. (Hemiptera: Aphididae) is a plant sap-sucking and virus-transmitting pest with high reproductive potential. It causes severe damage to multiple crops, such as beans and sugar beet, at infestation levels associated with meteorological changes and cultivars [1,2,3]. Due to a long-term dependence on chemical control, economically important aphid pests including *A. fabae* have evolved a diversity of biochemical and molecular mechanisms to resist or overcome toxic effects of chemical insecticides, such as neonicotinoids applied worldwide [4,5]. The enormous side effects of this dependence have led to post-neonicotinoid aphid control in Europe, where neonicotinoid insecticides have been banned for use in pest control [6]. The prohibition of neonicotinoid usage in Europe and its restriction on other continents makes it necessary to emphasize biological and cultural measures as alternative or complementary strategies for sustainable control of *A. fabae* and plant viruses which the aphid pest transmits [7,8].

Predators, parasitoids, and entomopathogenic fungi are well-known agents for biocontrol of aphid pests. Their compatibility and complementary effects on aphid control are attractive to researchers. Fungal virulence to *A. fabae* varies at inter- and intraspecific levels [9]. Co-application of aphid pathogens (*Lecanicillium muscarium*) and predators (*Adalia bipunctata*) has exhibited additive effects in reducing *A. fabae* populations [10]. The aphid predator *Hippodamia variegata* (Coccinellidae) showed an ability to avoid *A. fabae* infected by *Beauveria bassiana* on plants, suggesting a negligible fungal threat to the predator [11]. The *A. fabae* population on greenhouse plants was more effectively controlled by the spray of a 10^7^ conidia mL^−1^ suspension than the release of five coccinellid adults or two parasitoid adults per m^−2^ [12]. Combined application of *Metarhizium anisopliae* and pyrethrum was more efficacious against *A. fabae* than the fungus or the botanical chemical applied alone [13]. In addition, the actinobacterial strain *Streptomyces griseoflavus* PAL114 formulated in talc powder has also proved virulent against *A. fabae* [14]. However, the second-generation fecundity of *A. fabae* on plants endophytically colonized by *B. bassiana* via seed inoculation or leaf spray was significantly enhanced compared with that on uninoculated plants, although the first-generation fecundity was unaffected, suggesting that caution is needed for making use of multi-trophic interactions in aphid management [15]. Compared with the easy production and formulation of conidia as active agents of fungal formulations, aphid predators and parasitoids have scarcely been commercialized for field release when needed, although they can be augmented to improve aphid control by establishing banker plant or conservation biocontrol systems [16,17].

In addition to biological measures, increasing crop diversity via intercropping and growing resistant varieties are important cultural measures against aphid pests. The population density of *A. fabae* in three rows of faba bean intercropping with one row of dragonhead was significantly lower than that in the monocrop with the same level of fertilizer [18]. French beans grown with the border crop wheat or sunflower and sprayed with *M. anisopliae* showed a marked reduction in either *A. fabae* population density or damage severity and an encouraging increase in marketable yield compared with the control [19]. The adult size/weight, embryo count, and nymphal survival rate of *A. fabae* differed significantly among five bean cultivars, indicating the importance of resistant varieties for aphid control [20]. Interestingly, volatile organic compounds emitted by virus-infected bean plants may repel the vector *A. fabae*, suggesting viral transmission promoted by aphid migration from infected plants [21]. The resistance of sugar beet to *A. fabae* was proved to be predominately phloem-located [22]. While advanced genotyping tools available for identifying key genes involved in faba bean traits promote the screening of resistant lines that lower the fecundity and intrinsic increase rate of *A. fabae* populations [23], varieties with desired resistance against *A. fabae* remain scarce.

Despite a diversity of entomopathogenic fungi, the wide-spectrum entomopathogens *B. bassiana* and *M. anisopliae* serve as the major sources of fungal insecticides and acaricides for arthropod pest control worldwide [24,25]. Previously, fungal formulations in the form of emulsifiable oil suspension (ES) or wettable powder (WP) were applied at the rates of 2.5 × 10^13^ to 5.0 × 10^13^ conidia ha^−1^ for field control of aphids and whiteflies [26,27,28]. Such application rates represent very high costs for pest control and hence are hardly acceptable for growers. As mass production and formulation technologies continue to advance, the cost of fungal application has greatly reduced. For example, *B. bassiana* ZJU435 ES and *M. anisopliae* CQ421 ES, registered as wide-spectrum mycoinsecticides in China, were applied at the rates of no more than 1.5 × 10^13^ conidia ha^−1^ for effective control of major rice insect pests [29,30,31,32,33] and cereal aphids [34,35]. Particularly, *B. bassiana* ZJU435 ES applied biweekly at the mentioned rate was as efficacious as 10% imidacloprid WP sprayed biweekly at its label rate for seasonal control of cereal aphids at the booting to mid-milky stages of winter wheat; reducing the fungal rate to 1.0 × 10^13^ conidia ha^−1^ also resulted in a desirable efficacy against cereal aphids [34]. Importantly, *B. bassiana* ZJU435 ES is significantly more efficacious for field control of cereal aphids than *M. anisopliae* CQ421 ES irrespective of being applied at either of the aforementioned rates, because ovoid *B. bassiana* conidia are smaller than tubular *M. anisopliae* conidia and hence significantly more deposited on wheat plant surfaces when sprayed at either rate [35]. The different deposition rates suggest a superiority of *B. bassiana* ZJU435 to *M. anisopliae* CQ421 for the control of pests feeding on plant surfaces. However, the tested application rates still represent considerably high costs, which restrain extensive application of *B. bassiana* ZJU435 for aphid control. It is necessary to explore an effective strategy for sustainable aphid control through combined application of fungal and chemical insecticides to reduce the costs of fungal application and the chemical side effects.

Previously, the mortality trends of two aphid species (*Macrosiphoniella sanborni* and *Myzus persicae*) in response to aqueous dilutions of *B. bassiana* ES sprayed alone or in combination with imidacloprid at sublethal rates of 0.01–0.5 a.i. µg mL^−1^ showed remarkable synergistic effects [36], suggesting exploitability of the fungal–chemical interaction for aphid control. Despite negligible impact on aphid mortality, exposure to sublethal chemical rates physiologically weakened aphids and made them more vulnerable to fungal infection. At low (LD_30_) and sublethal (LD_10_) doses, imidacloprid was shown to have a very limited impact on the intrinsic increase rate and the net reproduction rate of an aphid predator (coccinellid) population [37]. Presently, neonicotinoid insecticides, such as imidacloprid, are still allowed for application for aphid control in China [38,39,40,41]. However, the synergistic effects of fungal–chemical combinations against aphid pests have not been sufficiently explored under field conditions. This field study sought to test whether differentially reduced rates of *B. bassiana* ZJU435 ES applied in combination with 1/5 of imidacloprid at the label rate provided effective or desirable control efficacies against *A. fabae*, which has evolved a high tolerance to low temperatures and frequently infests overwintered faba bean crops in spring, a season with frequent drizzle and warming weather in subtropical Zhejiang Province, China. Our goal aimed at unveiling how this fungal formulation can be applied at largely reduced costs for acceptable aphid control when combined with the chemical at a low rate to minimize side effects on agroecosystems and environments.

## 2. Materials and Methods

### 2.1. Fungal and Chemical Insecticides

The formulation *B. bassiana* ZJU435 ES (1 × 10^10^ conidia mL^−1^), registered as a wide-spectrum mycoinsecticide and produced by Greenation Bioengineering Co. (Chongqing, China), was used in this field study. The chemical insecticide applied in combination with the fungal formulation was 10% imidacloprid WP (Jiangsu Pesticides Institute Co., Nanjing, China), purchased from a local pesticide market.

### 2.2. Faba Bean Crop

**Figure 1:**
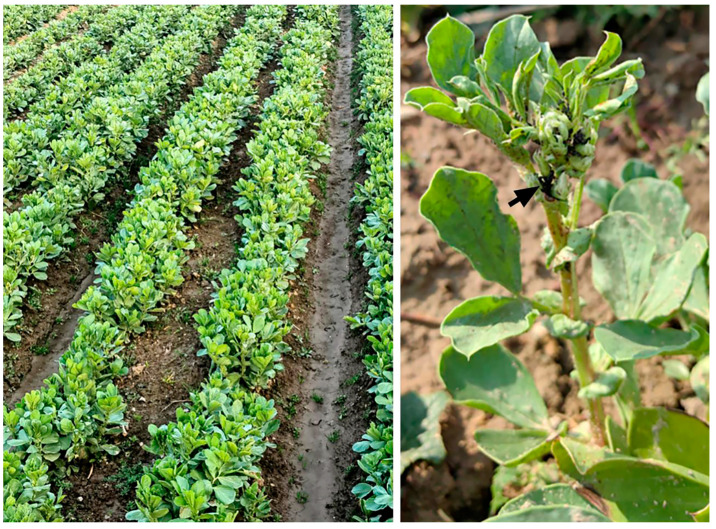
The faba bean crop located for initiation of field trials at Laishuitang Village, Qiaotou Town, Cixi City, Zhejiang. Left and right images show the budding stage of overwintered crop used for field trials in early spring and *A. fabae* (arrowed) damage to the tender shoots and buds of a plant, respectively.

Two fields (~0.3 ha each, ~600 m distant from each other) of faba bean (*Vicia faba* var. Dabaican) crop seeded in the farming area of Laishuitang Village (30°11′18.462″ N, 119°20′49.392″ E), Qiaotou Town, Cixi City, Zhejiang, on 24 October 2023 were identified for field trials in mid-February 2024. When located, the faba bean crop was at the stage of bud formation (left in Figure 1) and was heavily infested by *A. fabae*, which were feeding on tender shoots and buds (right in Figure 1). High-value fresh beans produced by these crops are usually harvested for local vegetable markets in April.

### 2.3. Setting up Field Trials

Each of the fields identified for repeated field trials (Trials 1 and 2) was divided into 18 plots (5 m × 10 m each) with edge and between-plot buffers of 1.5–2 m width. A unifactorial experiment design was adopted for the block-randomized arrangement of six treatments in the 18 plots, with three plots per treatment. The six treatments were the blank control, *B. bassiana* ZJU435 ES applied at the standard rate of 1.5 × 10^13^ conidia ha^−1^ (designated Bb), 10% imidacloprid WP applied at its label rate of 450 g ha^−1^ (designated ImD), and reduced fungal rates of 1.0 × 10^13^, 0.75 × 10^13^, and 0.5 × 10^13^ conidia ha^−1^ co-applied with 1/5 (90 g ha^−1^) of the chemical label rate (designated 2/3Bb + 1/5ImD, 2/3Bb + 1/5ImD, and 1/3Bb + 1/5ImD), respectively. Since green agriculture programs in China advocate 70% or more reduction in chemical application, 1/5 of the chemical label rate (80% decrease) was chosen for assessment of field interactions with the reduced application rates of *B. bassiana* used in this study.

The field trials under growers’ conventional management began with assessment of initial aphid population density on the morning of 19 February 2024, followed by the first spray in the afternoon of the same day. For the treatments, each plot was sprayed with 3 L of water (control), 3 L of water containing 7.5 mL of *B. bassiana* ZJU435 ES (Bb), 3 L of water containing 2.25 g of 10% imidacloprid WP (ImD), or 3 L of water containing 5.0, 3.75, or 2.5 mL (2/3, 1/2 or 1/3 Bb) of *B. bassiana* ZJU435 ES and 0.9 g (1/5 ImD) of 10% imidacloprid WP. The spraying was carried out using a 12 V lithium battery-driven knapsack airblast sprayer at a power of 25 W (Lanyi ScienTech Co., Shengzhen, China) and repeated biweekly during the six-week period of field trials.

### 2.4. Sampling of Aphid Density for Control Efficacy

The initial population density of *A. fabae* was assessed by counting the total number of nymphs and adults in situ for each of 20 samples, which included three plants grown from three seeds per hole that were randomly located in each of three plots per treatment. From then on, the sampling was repeated weekly to monitor changes in the aphid density (no. aphids per plant) in each treatment versus control per trial.

Aphid density data collected on each sampling occasion in Trial 1 or 2 were used to estimate the percent efficacy (%) of each treatment against the aphid pest, using the formula [1 − (*D*_t*j*_*D*_c0_/*D*_t0_*D*_c*j*_)] × 100. In the formula, *D*_c0_ and *D*_t0_ denote initial aphid densities in the control and each treatment, respectively, while *D*_c*j*_ and *D*_t*j*_ represent aphid densities in the control and each treatment on the *j*th day after the first spray, respectively.

### 2.5. Weather Data

Since fungal action on target pests depends to a large extent on environmental factors [42], weather data during the six-week period of field trials were requested from a local weather station. The weather records included daily temperature (mean, maximum and minimum), daily rainfall (mm), daily air humidity (%), and daily cloud level (%).

### 2.6. Statistical Analysis

All aphid density data and percent efficacies were normalized to logarithms and arcsine square roots, respectively. Variations in aphid density among the treatments in each trial on each sampling occasion was revealed through one-way analysis of variance (ANOVA), followed by Tukey’s honestly significant difference (HSD) test to assess differences between one treatment and another. Due to similar aphid densities present in each treatment in the two fields, normalized efficacy data for the treatments in two field trials were pooled together (6 observations per treatment) for one-way ANOVA and Tukey’s test on each sampling occasion. The normalized efficacy data from Trials 1 and 2 were further pooled for two-way ANOVA to compare overall efficacies of all treatments over the period of six weeks (sampling occasions pooled for treatment effect: 36 observations per treatment) and on each sampling occasion (treatments pooled for time effect: 30 observations per occasion) after the first spray, respectively.

## 3. Results

### 3.1. Weather Changes During Field Trials

**Figure 2:**
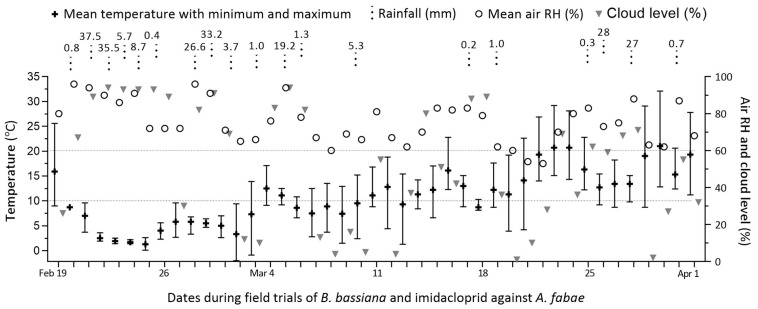
Weather records over the six-week period of field trials with *B. bassiana* and imidacloprid co-applied for aphid control on faba bean crops in Qiaotou, Cixi, Zhejiang.

Over the period of 42 days from early to late spring, the local weather increasingly warmed up, with 13 rainy days during the first three weeks and 6 rainy days during the last three weeks (Figure 2).

The records of daily mean temperatures presented weekly means (±SD) of 3.87 (±2.89), 6.46 (±2.92), 9.26 (±1.52), 11.91 (±2.47), 16.37 (±3.97), and 16.30 (±3.39) °C, respectively, across the six weeks of field trials. Particularly, the first two weeks of weather after the initial warm day featured high humidity and cloud levels but low temperatures, which were apparently unsuitable for the fungal action on aphids in the early spring.

### 3.2. Suppression of Aphid Population

**Figure 3:**
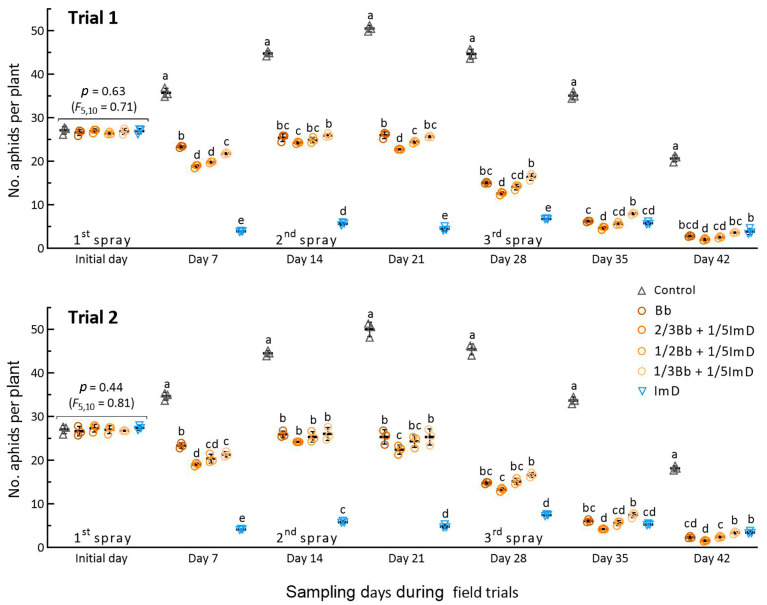
Suppression of *A. fabae* population by *B. bassiana* ZJU435 ES and 10% imidacloprid WP, which were applied alone at recommended rates (Bb and ImD) and co-applied at reduced rates in two field trials. The co-application rates were 2/3Bb, 1/2Bb, and 1/3Bb Bb plus 1/5ImD (2/3Bb + 1/5ImD, 1/2Bb + 1/5ImD, and 1/3Bb + 1/5ImD). Each field trial was sprayed biweekly over the six weeks. Different lowercase letters denote significant differences at *p* < 0.05 (Tukey’s test) among the treatments in each trial on each sampling day. Error bar: SD of the mean from three 50 m^2^ plots per treatment.

The mean (±SD) population densities of *A. fabae* on the initial day were 26.9 (±1.7) and 27.1 (±2.2) aphids per plant in Trials 1 and 2, respectively, with an insignificant variation in the treatments for the two trials (*p* ≥ 0.44 in *F*_5,10_ test). As illustrated in Figure 3, the aphid density in the control increased steadily during the first three weeks after the first spray and peaked at ~50 aphids per plant on day 21, followed by a gradual decrease to ~20 aphids per plant on day 42. The population increase in the control demonstrated a high tolerance of *A. fabae* to the low-temperature weather in early spring.

The aphid population densities in the Bb and Bb + ImD treatments over the first three-week period were significantly lower than those in the control but higher than those in the ImD treatment, indicating the slow action of *B. bassiana* on the aphid pest and the high aphidicidal activity of imidacloprid at low temperatures in early spring. As the weather subsequently warmed up, the Bb and Bb + ImD treatments increasingly suppressed the aphid populations after the second and third sprays. On most sampling occasions, the treatment 2/3Bb + 1/5 ImD reduced the aphid density significantly more than the Bb and other Bb + ImD treatments. Particularly, the third spray greatly reduced the differences in aphid densities among the treatments, suggesting an elevation of fungal aphidicidal activity with increasing temperature in the last two weeks of late spring. These observations indicated that the reduced rates of *B. bassiana* ZJU435 ES in combination with 1/5 of imidacloprid label rate were effective in suppressing the increase in aphid population in spring.

### 3.3. Efficacies of Different Treatments Against A. fabae

**Figure 4:**
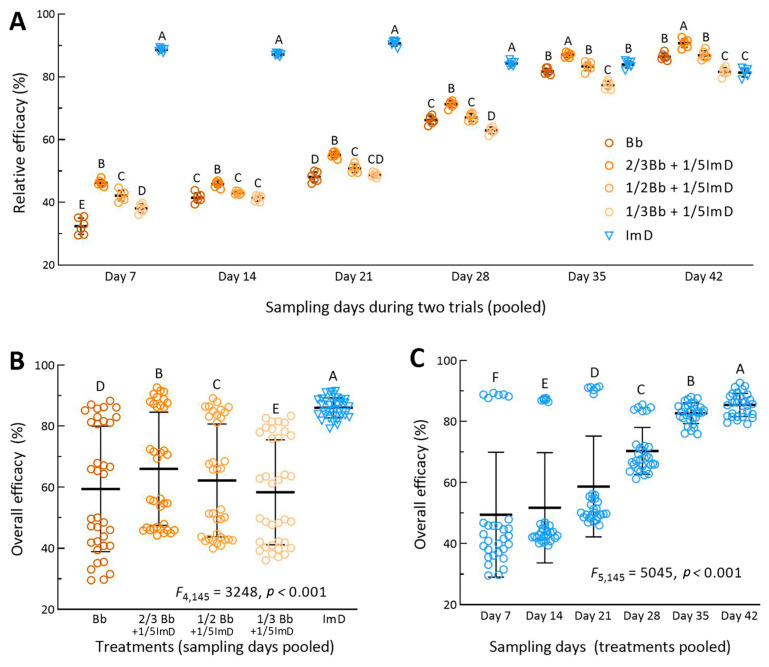
Relative efficacies of *B. bassiana* ZJU435 ES applied alone at recommended rate (Bb) and at reduced rates (2/3Bb, 1/2Bb, and 1/3Bb) plus 1/5ImD for control of *A. fabae* populations on faba bean crops. (**A**) Relative efficacies of different treatments pooled by sampling occasions from two field trials (*n* = 6 per treatment for one-way ANOVA). (**B**,**C**) Treatment effect (*n* = 36 per treatment) and time effect (*n* = 30 per sampling occasion) revealed by two-way ANOVA of all relative efficacies pooled over the six-week period for the two field trials. Different uppercase letters indicate significant differences at *p* < 0.01 (Tukey’s test). Error bar: SD of the mean from 6 (**A**), 36 (**B**), and 30 (**C**) observations.

For each of the treatments, the aphid population densities in the two fields were similar on all sampling occasions. For this reason, the percent efficacies of each treatment in Trials 1 and 2 were pooled for analysis of overall effect. One-way ANOVA revealed different efficacies of all treatments on each of six sampling occasions after the first spray. The standard Bb treatment resulted in a mean (±SD, *n* = 6) efficacy of 32.5% (±2.6) on day 7, 41.5% (±1.5) on day 14, 48.1% (±1.6) on day 21, 66.3% (±1.3) on day 28, 81.8% (±1.0) on day 35, and 86.5% (±1.1) on day 42 (Figure 4A). These efficacies were significantly lower than those for the 2/3Bb + 1/5ImD treatment (*p* < 0.01 in Tukey’s test) and more or less different from those for other Bb + ImD treatments. The efficacy of the standard chemical treatment was consistently highest (84.3–90.7%) on days 7–28 but significantly lower than that in the 2/3Bb + 1/5ImD treatment and lower than or close to those observed in the Bb and other Bb + ImD treatments on day 35 or 42.

Two-way ANOVA of all efficacy data pooled from the treatments of two field trials over six sampling occasions revealed very high significances of treatment effect (*F*_4,145_ = 3248, *p* < 0.0001) and time effect (*F*_4,145_ = 5045, *p* < 0.0001) and also a high significance of interactive effect between the two factors (*F*_20,145_ = 462, *p* < 0.0001). The overall mean (±SD, *n* = 36) efficacy was of 86.0% (±3.3) for the chemical treatment, followed in the order 66.0% (±18.5), 62.2% (±18.5), 59.4% (±20.6), and 58.4% (±17.2) for the treatments 2/3Bb + 1/5ImD, 1/2Bb + 1/5ImD, Bb, and 1/3Bb + 1/5ImD (Figure 4B), respectively. The overall mean efficacies differed significantly from one treatment to another (*p* < 0.01 in Tukey’s test). The mean efficacy (*n* = 30) of all five treatments in the two trials was 49.5% (±20.5) on day 7, 51.8% (±18.1) on day 14, 58.7% (±16.5) on day 21, 70.4% (±7.6) on day 28, 86.7% (±3.4) on day 35, and 85.3% (±3.8) on day 42 (Figure 4C). The increase in the mean efficacy and the decrease in its standard deviation with increasing days in the field trials demonstrated that the aphidicidal activity of *B. bassiana* in the Bb and Bb + ImD treatments increased with the warming weather during the six-week period of the field trials and increasingly approached that of the chemical applied alone at its label rate.

## 4. Discussion

In Cixi, Zhejiang, overwintered faba bean crops at the stage of bud formation were heavily infested by *A. fabae* population in the spring, with a wide range of temperature fluctuation. The first spraying was followed by three weeks of low-temperature weather at weekly means of 3.87, 6.46, and 9.26 °C, with a population increase to a peak of ~50 aphids per plant in the control. This indicates a high tolerance of local *A. fabae* population to low temperatures in early spring. Previously, obligate endosymbiont bacterium (*Buchnera aphidicola*) populations were suppressed in *A. fabae* briefly exposed to sublethal heat at 38 °C, leading to increased mortality, delayed development, and reduced fecundity of the aphid host during a 5-day period of recovery at 20 °C [43]. This report suggests a likelihood that the high tolerance of *A. fabae* in Zhejiang to low temperatures could be associated with the prosperity of its endosymbiont population in early spring, warranting further study for confirmation.

The unexpected low-temperature weather during the first three weeks, with frequent drizzle, was apparently unsuitable for the action of *B. bassiana* on the *A. fabae* population in the field, since the optimal temperature for conidial germination of the fungus and its hyphal growth and invasion into the insect host is around 25 °C [44]. For this reason, the population density of *A. fabae* during the period was greatly reduced by the treatment with imidacloprid at the label rate rather than the four treatments with *B. bassiana* at the recommended rate and at reduced rates plus 1/5 of the chemical label rate. Interestingly, the four treatments effectively hindered the increase in *A. fabae* population observed in the control, indicating that such treatments had slow suppressive effects on the aphid population even at low temperatures. As the subsequent weather was warming up, the four treatments played increasing roles in reducing the aphid density on the crop after the second and third sprays. Their mean efficacies against *A. fabae* in the two field trials after the third spray reached 77–87% on day 35 and 82–91% on day 42. These efficacies were higher than or close to those achieved with the chemical treatment in this study and the previous mean efficacy of 84% or 85% for the same fungal formulation applied at a rate of 1.5 × 10^13^ conidia ha^−1^ for field control of cereal aphids during a warmer period (April 7 to May 12 in 2023) in Yuhang, Hangzhou, Zhejiang [34,35]. The overall mean efficacies against *A. fabae* over the six-week period of the two field trials demonstrated that the treatments 2/3Bb + 1/5ImD and 1/2Bb + 1/5ImD were superior to the treatment with *B. bassiana* applied at the recommended rate, which was only 1% more efficacious than the 1/3Bb + 1/5ImD treatment. These results provide direct field evidence of the synergistic effects of fungal–chemical interaction against insect pests, as previously revealed in laboratory studies [36,45,46].

Moreover, the costs of *B. bassiana* ZJU435 ES and 10% imidacloprid WP applied at recommended rates for pest control are USD ~60 and ~5 ha^−1^, respectively, based on their market prices in China. The three fungus–chemical combinations tested in this field study reduced the application costs to USD ~41, ~31, and ~21 ha^−1^, representing cost reductions of 31.7%, 48.3%, and 65.0%, respectively, in comparison to the fungal formulation applied alone at the recommended rate. The relatively high efficacies of these fungus–chemical combinations against *A. fabae* at reduced cost indicate that utilizing the synergistic effects of fungal–chemical interactions for pest control is a promising strategy. Extensive applications of fungal insecticides still incur high costs, based on current technologies.

Notably, the overall mean efficacies of all combined treatments were obviously affected by the low-temperature weather during the first three weeks of this field study. This indicates the importance of weather data for the analysis of fungal efficacy against aphid pests in the field and highlights the environmental impact of the insecticidal activity of fungal formulations [42]. By contrast, changes in air humidity over the period of the field trials appeared to be less influential on the fungal efficacy of the treatments. This is because the dependence of conidial germination on high humidity can be greatly reduced by oils used in the conidial formulation [47] and nearly abolished by emulsifiable oil-formulated *B. bassiana* conidia incubated at the regimes of 20–30 °C and 51–95% RH [48]. Additionally, air humidity as reflected by the weather records is obviously lower than under the crop canopy. We infer that the overall mean efficacies of fungus–chemical combinations against aphids and other insect pests can be enhanced in more suitable seasons, warranting more field studies.

Finally, it remains unclear whether the sublethal rates of imidacloprid applied in combination with the reduced application rates of *B. bassiana* affected aphid predators and parasitoids. Indeed, common aphid predators and parasitoids were rarely observed in these field trials, perhaps due to the season being too early for their activities. Previously, the impact of *B. bassiana* infection on aphid predators proved negligible since the predators were able to avoid the aphids infected by the fungus [10]. LD_10_ and LD_30_ of imidacloprid were revealed to affect the reproduction potential of aphid predators at limited levels [37]. Based on previous reports, the non-target effect of 1/5 of the imidacloprid label rate applied together with *B. bassiana* in this study may have been very limited if it existed. Meanwhile, major aphid pests have developed different levels of resistance to neonicotinoids, including imidacloprid [4,41,49]. Such chemicals prophylactically applied for pest control have widely accumulated in waterways, soils, field margin plants, and floral resources, threatening environmental safety [50]. Thus, cautious strategies are needed for chemicals that are still permitted for pest control application in China [39,40,41] but banned for use in Europe [6]. Sublethal and low rates of these chemicals applied for synergistic fungus–chemical interactive effects against insect pests are apparently in accordance with a cautious strategy to largely mitigate their side effects on agroecosystems and environments.

In conclusion, *B. bassiana* ZJU435 ES effectively protected overwintered faba bean crops in Zhejiang, China from *A. fabae* damage from early to late spring. Its efficacy against the aphid pest increased with the warming weather throughout the spring and was further enhanced at 1/2 to 2/3 of its recommended rate co-applied with 1/5 of imidacloprid at the label rate. Notably, the treatment with 1/3 of its recommended rate plus 1/5 of imidacloprid at the label rate resulted in an overall mean efficacy only 1% lower than that ascribed to the fungal formulation applied alone at the recommended rate. The fungal–chemical combinations against the aphid pest reduced the fungal application cost by 32–65%, representing a low-cost–high-efficacy strategy in the context of the extensive application of *B. bassiana* ZJU435 ES, which is still expensive for growers. Sublethal application rates of imidacloprid incorporated into these combinations may largely mitigate the side effects of the chemical on agroecosystems and environments. Therefore, combined applications of fungal and chemical insecticides at largely reduced rates to obtain synergistic effects of fungal–chemical interactions are alternative strategies applicable for the sustainable control of insect pests on crops.

## Data Availability

The original contributions presented in this study are included in the article. Further inquiries can be directed to the corresponding authors.

## References

[1] Akca I., Ayvaz T., Yazici E., Smith C.L., Chi H. (2015). Demography and population projection of *Aphis fabae* (Hemiptera: Aphididae): With additional comments on life table research criteria. J. Econ. Entomol..

[2] Shih P.Y., Sugio A., Simon J.C. (2023). Molecular mechanisms underlying host plant specificity in aphids. Annu. Rev. Entomol..

[3] Almogdad M., Lavrukaite K., Semaskiene R. (2024). Temporal analysis of the relationship between black bean aphid (*Aphis fabae*) infestation and meteorological conditions in faba bean (*Vicia faba*). Agronomy.

[4] Edwards O.R., Franzmann B., Thackray D., Micic S. (2008). Insecticide resistance and implications for future aphid management in Australian grains and pastures: A review. Aust. J. Exp. Agric..

[5] Bass C., Nauen R. (2023). The molecular mechanisms of insecticide tesistance in aphid crop pests. Insect Biochem. Mol. Biol..

[6] Mc Namara L., Gauthier K., Walsh L., Thebaud G., Gaffney M., Jacquot E. (2020). Management of yellow dwarf Disease in Europe in a post-neonicotinoid agriculture. Pest Manag. Sci..

[7] Ndakidemi B.J., Mbega E.R., Ndakidemi P.A., Stevenson P.C., Belmain S.R., Arnold S.E.J., Woolley V.C. (2021). Natural pest regulation and its compatibility with other crop protection practices in smallholder bean farming systems. Biology.

[8] Francis F., Then C., Francis A., Gbangbo Y.A.C., Iannello L., Ben Fekih I. (2022). Complementary strategies for biological control of aphids and related virus transmission in sugar beet to replace neonicotinoids. Agriculture.

[9] Qubbaj T., Samara R. (2022). Efficacy of three entomopathogenic fungi *Beauveria bassiana*, *Metarhizium anisopliae* and *Lecanicillium lecanii* Isolates against black bean aphid, *Aphis fabae* (Scop.) (Hemiptera: Aphididae) on faba bean *(Vicia faba* L.). Legume Res..

[10] Mohammed A.A. (2018). *Lecanicillium muscarium* and *Adalia bipunctata* combination for the control of black bean aphid, *Aphis fabae*. BioControl.

[11] Seiedy M., Heydari S., Tork M. (2015). Orientation of *Hippodamia variegata* (Coleoptera: Coccinellidae) to healthy and *Beauveria bassiana*-Infected *Aphis fabae* (Hemiptera: Aphididae) in an olfactometer system. Turk. J. Zool..

[12] Nordey T., Boni S.B., Agbodzavu M.K., Mwashimaha R., Mlowe N., Ramasamy S., Deletre E. (2021). Comparison of biological methods to control *Aphis fabae* Scopoli (Hemiptera: Aphididae) on kalanchoe crops in East Africa. Crop Prot..

[13] Fernández-Grandon G.M., Harte S.J., Ewany J., Bray D., Stevenson P.C. (2020). Additive effect of botanical insecticide and entomopathogenic fungi on pest mortality and the behavioral response of its natural rnemy. Plants.

[14] Benbelkhir F.Z., Allali K., Benadjila A., Goudjal Y., Medjekal S., Zamoum M. (2024). Development of bioinsecticide based on *Streptomyces griseoflavus* PAL114 for control of black bean aphids *Aphis fabae*. Biocontrol Sci. Technol..

[15] Jensen R.E., Enkegaard A., Steenberg T. (2019). Increased fecundity of *Aphis fabae* on *Vicia faba* plants following seed or leaf inoculation with the entomopathogenic fungus *Beauveria bassiana*. PLoS ONE.

[16] Frank S.D. (2010). Biological control of arthropod pests using banker plant systems: Past progress and future directions. Biol. Control.

[17] Pell J.K., Hannam J.J., Steinkraus D.C. (2010). Conservation biological control using fungal entomopathogens. BioControl.

[18] Azimi S., Amini R. (2015). Population density of *Aphis fabae* Scopoli (Hemiptera, Aphididae) and its natural enemies in intercropping of faba bean (*Vicia faba* L.) and dragonhead (*Dracocephalum moldavica* L.). J. Biodivers. Environ. Sci..

[19] Emaru A., Nyaanga J.G., Saidi M. (2024). Integrating *Metarhizium anisopliae* entomopathogenic fungi with border cropping reduces black bean aphids (*Aphis fabae*) damage and enhances yield and quality of French bean. Heliyon.

[20] Meradsi F., Laamari M. (2016). Population dynamics and biological parameters of *Aphis fabae* Scopoli on five broad bean cultivars. Int. J. Biosci..

[21] Wamonje F.O., Tungadi T.D., Murphy A.M., Pate A.E., Woodcock C., Caulfield J.C., Mutuku J.M., Cunniffe N.J., Bruce T.J.A., Gilligan C.A. (2020). Three aphid-transmitted viruses encourage vector migration from infected common bean (*Phaseolus vulgaris*) plants through a combination of volatile and surface cues. Front. Plant Sci..

[22] Zhu Y.S., Stahl A., Rostás M., Will T. (2024). Temporal and species-specific resistance of sugar beet to green peach aphid and black bean aphid: Mechanisms and implications for breeding. Pest Manag. Sci..

[23] Skovgård H., Stoddard F.L. (2023). Reproductive potential of the black bean aphid (*Aphis fabae* Scop.) on a range of faba bean (*Vicia faba* L.) accessions. Legume Sci..

[24] de Faria M., Wraight S.P. (2007). Mycoinsecticides and mycoacaricides: A comprehensive list with worldwide coverage and international classification of formulation types. Biol. Control.

[25] Arthurs S., Dara S.K. (2019). Microbial Biopesticides for invertebrate pests and their markets in the United States. J. Invertebr. Pathol..

[26] Poprawski T.J., Parker P.E., Tsai J.H. (1999). Laboratory and field evaluation of hyphomycete insect pathogenic fungi for control of brown citrus aphid (Homoptera: Aphididae). Environ. Entomol..

[27] Vandenberg J.D., Sandvol L.E., Jaronski S.T., Jackson M.A., Souza E.J., Halbert S.E. (2001). Efficacy of fungi for control of Russian wheat aphid (Homoptera: Aphididae) in irrigated wheat. Southwest Entomol..

[28] Wraight S.P., Carruthers R.I., Jaronski S.T., Bradley C.A., Garza C.J., Galaini-Wraight S. (2000). Evaluation of the entomopathogenic fungi *Beauveria bassiana* and *Paecilomyces fumosoroseus* for microbial control of the silverleaf whitefly, *Bemisia argentifolii*. Biol. Control.

[29] Tang J.F., Liu X.Y., Ding Y.C., Jiang W.J., Xie J.Q. (2019). Evaluation of *Metarhizium anisopliae* for rice planthopper control and its synergy with selected insecticides. Crop Prot..

[30] Peng G.X., Xie J.Q., Guo R., Keyhani N.O., Zeng D.Y., Yang P.Y., Xia Y.X. (2021). Long-term field evaluation and large-scale application of a *Metarhizium anisopliae* strain for controlling major rice pests. J. Pest Sci..

[31] Peng Y.F., Tang J.F., Hong M.S., Xie J.Q. (2020). Suppression of rice planthopper populations by the entomopathogenic fungus *Metarhizium anisopliae* without affecting the rice microbiota. Appl. Environ. Microbiol..

[32] Qi D.Y., Xu W.Y., Shao Y.Z., Feng J.R., Feng M.G., Tong S.M. (2023). Mycoinsecticides applied in late afternoon are more efficacious against rice leaf-rolling insect pests than those in the morning. Biol. Control.

[33] Xu W.Y., Wen Z.X., Li X.J., Hu E.Z., Qi D.Y., Feng M.G., Tong S.M. (2023). Timing of fungal insecticide application to avoid solar ultraviolet irradiation enhances field control of rice planthoppers. Insects.

[34] Qi D.Y., Shao Y.Z., Yang R., Liu C.L., Feng G.H., Pan W.Y., Feng M.G., Tong S.M. (2024). Emulsifiable oil-formulated *Beauveria bassiana* competes with imidacloprid for seasonal control of cereal aphids in Zhejiang, China. Pest Manag. Sci..

[35] Tong S.M., Qi D.Y., Liu C.L., Feng G.H., Pan W.Y., Shao Y.Z., Yang R., Feng M.G. (2024). Comparative efficacies of two fungal insecticides in seasonal control of cereal aphids heavily infesting winter wheat. BioControl.

[36] Ye S.D., Dun Y.H., Feng M.G. (2005). Time and concentration dependent interactions of *Beauveria bassiana* with sublethal rates of imidacloprid against the aphid pests *Macrosiphoniella sanborni* and *Myzus persicae*. Ann. Appl. Biol..

[37] Skouras P.J., Darras A.I., Mprokaki M., Demopoulos V., Margaritopoulos J., Delis C., Stathas G.J. (2021). Toxicity, sublethal and low dose effects of imidacloprid and deltamethrin on the aphidophagous predator *Ceratomegilla undecimnotata* (Coleoptera: Coccinellidae). Insects.

[38] Gao Z.S., Zhang X.F., Liu H.T., Zhang W.J., Mu W. (2016). Feasibility for controlling wheat aphids by seed dressing with neonicotinoid insecticides. Acta Phytophyl. Sin..

[39] Zhang P., Zhang X.F., Zhao Y.H., Wei Y., Mu W., Liu F. (2016). Effects of imidacloprid and clothianidin seed treatments on wheat aphids and their natural enemies on winter wheat. Pest Manag. Sci..

[40] Zhang Z., Li Y.P., Li X.R., Zhu X., Zhang Y.H. (2023). Efficacy of imidacloprid seed treatments against four wheat aphids under laboratory and field conditions. Plants.

[41] Xu T.Y., Zhang S., Liu Y., Ma L., Li X.Q., Zhang Y.X., Fan Y.J., Song D.L., Gao X.W. (2022). Slow resistance evolution to neonicotinoids in field populations of wheat aphids revealed by insecticide resistance monitoring in China. Pest Manag. Sci..

[42] Jaronski S.T. (2010). Ecological factors in the inundative use of fungal entomopathogens. BioControl.

[43] Zhang B., Leonard S.P., Li Y.Y., Moran N.A. (2019). Obligate bacterial endosymbionts limit thermal tolerance of insect host species. Proc. Natl. Acad. Sci. USA.

[44] Yu L., Xu S.Y., Luo X.C., Ying S.H., Feng M.G. (2024). High photoreactivation activities of Rad2 and Rad14 in recovering insecticidal *Beauveria bassiana* from solar UV damage. J. Photochem. Photobiol. B-Biol..

[45] Feng M.G., Pu X.Y. (2005). Time-concentration-mortality modeling of the synergistic interaction of *Beauveria bassiana* and imidacloprid against *Nilaparvata lugens*. Pest Manag. Sci..

[46] Tian L., Feng M.G. (2006). Evaluation of the time-concentration-mortality responses of *Plutella xylostella* larvae to the interaction of *Beauveria bassiana* with a nereistoxin analogue insecticide. Pest Manag. Sci..

[47] Malsam O., Kilian M., Oerke E.C., Dehne H.W. (2002). Oils for increased efficacy of *Metarhizium anisopliae* to control whiteflies. Biocontrol Sci. Technol..

[48] Shi W.B., Feng M.G., Liu S.S. (2008). Sprays of emulsifiable *Beauveria bassiana* formulation are ovicidal towards *Tetranychus urticae* (Acari: Tetranychidae) at various regimes of temperature and humidity. Exp. Appl. Acarol..

[49] Bass C., Denholm I., Williamson M.S., Nauen R. (2015). The global status of insect resistance to neonicotinoid insecticides. Pest. Biochem. Physiol..

[50] Goulson D. (2013). REVIEW: An overview of the environmental risks posed by neonicotinoid insecticides. J. Appl. Ecol..

